# Non-Isothermal Sublimation Kinetics of 2,4,6-Trinitrotoluene (TNT) Nanofilms

**DOI:** 10.3390/molecules24061163

**Published:** 2019-03-23

**Authors:** Walid M. Hikal, Brandon L. Weeks

**Affiliations:** 1 Department of Chemical Engineering, Texas Tech University, Lubbock, TX 79409, USA; brandon.weeks@ttu.edu; 2 Department of Mathematics, Australian College of Kuwait, Safat 13015, Kuwait; 3 Department of Physics, Faculty of science, Assiut University, Assiut 71516, Egypt

**Keywords:** 2,4,6-trinitrotoluene (TNT), sublimation, activation energy, UV spectroscopy, spin coating, explosives detection

## Abstract

Non-isothermal sublimation kinetics of low-volatile materials is more favorable over isothermal data when time is a crucial factor to be considered, especially in the subject of detecting explosives. In this article, we report on the in-situ measurements of the sublimation activation energy for 2,4,6-trinitrotoluene (TNT) continuous nanofilms in air using rising-temperature UV-Vis absorbance spectroscopy at different heating rates. The TNT films were prepared by the spin coating deposition technique. For the first time, the most widely used procedure to determine sublimation rates using thermogravimetry analysis (TGA) and differential scanning calorimetry (DSC) was followed in this work using UV-Vis absorbance spectroscopy. The sublimation kinetics were analyzed using three well-established calculating techniques. The non-isothermal based activation energy values using the Ozawa, Flynn–Wall, and Kissinger models were 105.9 ± 1.4 kJ mol^−1^, 102.1 ± 2.7 kJ mol^−1^, and 105.8 ± 1.6 kJ mol^−1^, respectively. The calculated activation energy agreed well with our previously reported isothermally-measured value for TNT nanofilms using UV-Vis absorbance spectroscopy. The results show that the well-established non-isothermal analytical techniques can be successfully applied at a nanoscale to determine sublimation kinetics using absorbance spectroscopy.

## 1. Introduction

Explosives detection keeps rising as a critical issue due to the global rise in terrorist activity and needs more intensive investigation. Sublimation kinetics of explosives at a submicron scale or even nanoscale is crucial for their trace detection [1]. Measuring the sublimation kinetics of an explosive in the nanometer scale could be used to determine its persistence as well as its lifetime. 2,4,6-trinitrotoluene (TNT) is among the most widely used secondary explosives in both military and industry applications due to its low sensitivity and safe handling. However, there is a large discrepancy in the activation energy values reported for TNT’s sublimation value in literature (90–141 kJ/mol).

Both isothermal and non-isothermal sublimation kinetics are often measured using thermogravimetry analysis (TGA), differential scanning calorimetry (DSC), and differential thermal analysis (DTA). Sublimation kinetics is usually measured using TGA where a flow of gas through both the balance chamber and the sample is often needed to prevent temperature and pressure build-up [2,3]. However, the mass change detectable by TGA (few nanograms) limits usefulness to samples larger than a few milligrams, depending on the sensitivity of the balance. This makes TGA an unreliable technique to study thermodynamic properties of nanofilms. Quartz crystal microbalance (QCM) [4,5] and atomic force microscopy (AFM) [6,7] have been used to isothermally determine sublimation kinetics of explosives’ microparticles. However, both techniques cannot be operated neither in-situ nor non-isothermally. AFM and QCM also require long times in data collection. This is expected to introduce errors to the measured sublimation kinetics.

Recently, we reported a new in-situ methodology to isothermally determine sublimation rates, activation energies of sublimation, and vapor pressures of continuous nanofilms of low volatile materials using UV-absorbance spectroscopy [8,9,10]. The determined sublimation rates were shown to be more accurate than those obtained using both AFM and QCM. Using UV-Vis absorbance spectroscopy in determining sublimation kinetics has the advantage of eliminating the surface area from the rate equation. In addition, the relatively small scanned area ensures accurate measurements even with the existence of surface roughness, dislocations, and cracks. The technique was shown to be accurate when operated isothermally. It was also shown that when operated non-isothermally using one set of data at a single heating rate, values within the reported data were achieved. 

In this article we illustrate the successful use of UV-Vis absorbance spectroscopy in non-isothermal measurement of TNT sublimation kinetics, using different heating rates and three different famous calculation techniques. TNT sublimation kinetics were only determined non-isothermally once before using UV-Vis spectroscopy with a crude temperature integral approximation in the kinetics equation used [11]. No other techniques have been reported to measure the sublimation kinetics of secondary explosives non-isothermally due their low melting point (80 °C for TNT). In addition, the common procedure used to determine sublimation kinetics non-isothermally in famous techniques uses different heating rates. However, this procedure is not suitable in these techniques for low volatile explosives such as TNT, 1,3,5-Trinitro-1,3,5-triazinane (RDX), and Pentaerythritol tetranitrate (PETN). For the first time, we report the use of this procedure to non-isothermally determine the activation energy of TNT sublimation using absorbance spectroscopy.

## 2. Experimental Section 

2,4,6-trinitrotoluene (TNT) was provided by Austin explosives and was purified by a crystallization technique using the evaporation methodology from acetone solution. The resulting rod-like pure crystallites were then dissolved in acetone at room temperature. A stock solution of 0.2 M TNT was used for preparing continuous TNT films on quartz substrates by spin coating (single wafer spin processor, Laurell technologies corp., North Wales, PA, USA) of 20 µL TNT solutions at 4500 rpm for one minute. The quartz substrates were cleaned using acetone and de-ionized water before the films were deposited.

The films were characterized by a PSIA XE AFM (Santa Clara, CA, USA) in contact mode with a silicon cantilever (Nanosensor pointprobes, Nominal spring constant 5.0 N/m). Absorbance of TNT was recorded in situ using a Lambda 1050 UV/Vis/NIR (Perkin-Elmer, UK) spectrometer at 1.0 nm resolution. The spectrometer is equipped with a temperature controller, with an accuracy of 0.05 °C, allowing for in situ temperature-dependent absorbance measurements for both sample and reference. Time-driven measurements of TNT absorbance at the peak with highest absorbance were collected at different heating rates, at different temperatures below the melting point (80 °C).

## 3. Theory

The dependence of kinetic process rates on temperature is usually represented by the temperature-dependent rate constant, *k(T)*, and the dependence on the extent of conversion by the reaction model, *f*(α). Sublimation kinetics of a solid volatile material is usually studied using thermogravimetric analysis (TGA) and is often described by the following basic equation:

(1) $$-\frac{dm}{dt}=k\left(T\right)f\left(\alpha \right)$$

where *m* is the mass, α is the extent of conversion which is defined as $\alpha =\frac{\left(w_{i}-w_{T}\right)}{\left(w_{i}-w_{f}\right)}$ (where $w_{i}$ and $w_{f}$ the initial and final mass, respectively, while $w_{T}$ is the mass loss at temperature *T*), *f*(α) is the model function, which assumes different mathematical forms depending on the reaction mechanism [12], and *k(T)* is the specific rate constant in g/s, whose temperature dependence is commonly described by the Arrhenius equation [13]:

(2) $$lnk\left(T\right)=lnA-E_{a}/RT$$

where $E_{a}$ is the activation energy, *A* is the pre-exponential/frequency factor, *R* the gas constant and *T* the absolute temperature. The plot of ln(k) versus 1/T is linear and from the slope, the activation energy ($E_{a}$) for the sublimation is calculated. The proposed kinetic analysis, based on dynamic model-free methods using data obtained at different fixed heating rates, $\beta =\frac{dT}{dt}$ seems to be the most reliable approach. The thermal sublimation kinetics is examined using the onset temperature and the Ozawa equation given by [14]:

(3) $$ln\left(\beta \right)=const-1.052\left(\frac{E_{a}}{RT_{\alpha }}\right)$$

Kissinger’s Technique was also used to determine the activation energy of sublimation using the equation [15,16,17]:

(4) $$ln\beta /T_{m}^{2}=ln\left(\frac{AR}{E_{a}}\left[n{\left(1-\alpha \right)}_{m}^{n-1}\right]\right)-\frac{E_{a}}{RT_{m}}$$

where $T_{m}$ is the 50% mass loss temperature at a given β value, A the pre-exponential factor, *E_a_* the activation energy and *R* the gas constant. The *E_a_* value can be calculated from the slope of $ln\left(\beta /T_{m}^{2}\right)$ as a function of $1/T_{m}$. Kinetic study of sublimation steps was also performed using the isoconversional method of Flynn–Wall Technique [18]. The isoconversional expression is,

(5) $$ln\left(\beta \right)=ln\left(\frac{AE_{a}}{R}\right)-lnF\left(\alpha \right)-E_{a}/RT$$

where F(α) is the integral form of f(α). The activation energy, *E_a_* can be calculated from a plot of *ln*β versus *1/T* at a fixed weight loss since the slope of such a line is given by *E_a_/R* (gas constant 8.314 J mol^−1^ K^−1^). The *lnA* is calculated from the intercept value of the line and the derived *E_a_* value.

In using absorbance spectroscopy to determine the sublimation kinetics, the sublimation kinetics can be determined by relating the mass loss to the decrease in the thickness of a nanofilm of the material, assuming a homogenous sublimation from the films’ surface as follows: for a nanofilm of volume V, surface area S, and thickness *l*, we have $\frac{dm}{Sdt}=\frac{\rho dV}{Sdt}=\frac{\rho dl}{dt}$, where ρ is the density of the material. The optical absorbance (*A*) of a nanofilm of thickness l and absorbance coefficient *α* is given by the Lambert law and can be written as [19,20] A=α l, thus the mass loss can be written as $\frac{\rho }{\alpha }\frac{dA}{dt}$. Hence the rate of the absorbance decrease can be used as property to monitor the mass loss and can be used to determine the sublimation kinetics.

## 4. Results and Discussion

TNT nanofilms used in this study have been shown to be continuous by using optical microscopy. In addition, the continuity of the films has been confirmed by atomic force microscopy. The thickness of the films was measured using AFM operated in contact mode at room temperature by removing a part of the film using a tape. The films thicknesses are ~500–600 nm with an RMS surface roughness of ~30 nm [8].

**Figure 1:**
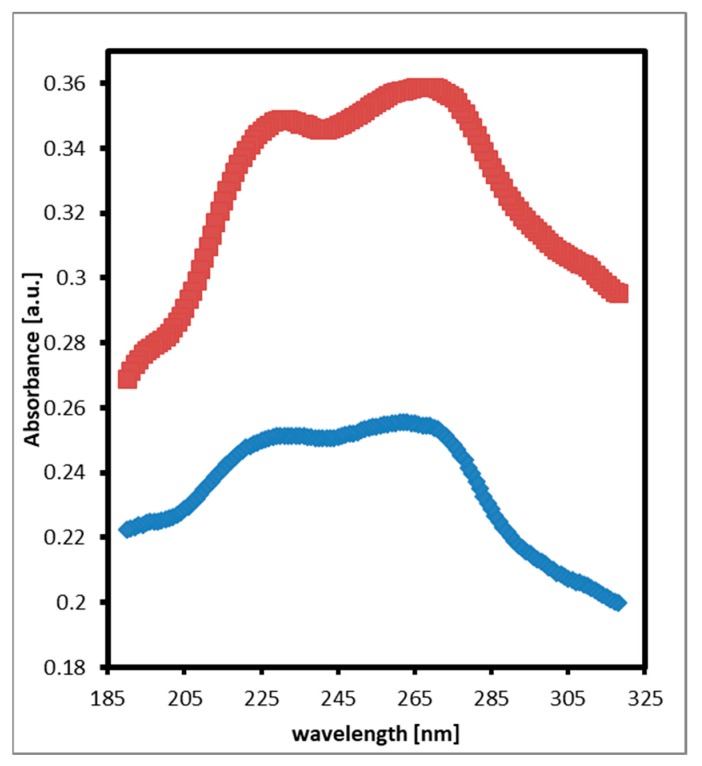
Absorbance spectrum of TNT nanofilms at 50 °C (bottom) and 70 °C (top) showing no shift in the absorbance peaks upon temperature change.

TNT nanofilms exhibit two prominent absorbance peaks centered at 264 nm and 230 nm as shown in Figure 1. However, the two peaks are broad and overlap. The location of the absorbance peaks was determined by the second derivatives of the absorbance spectra using UV Winlab Data Processor & Viewer software. The locations of the two prominent peaks do not change over the temperature range used in this work.

**Figure 2:**
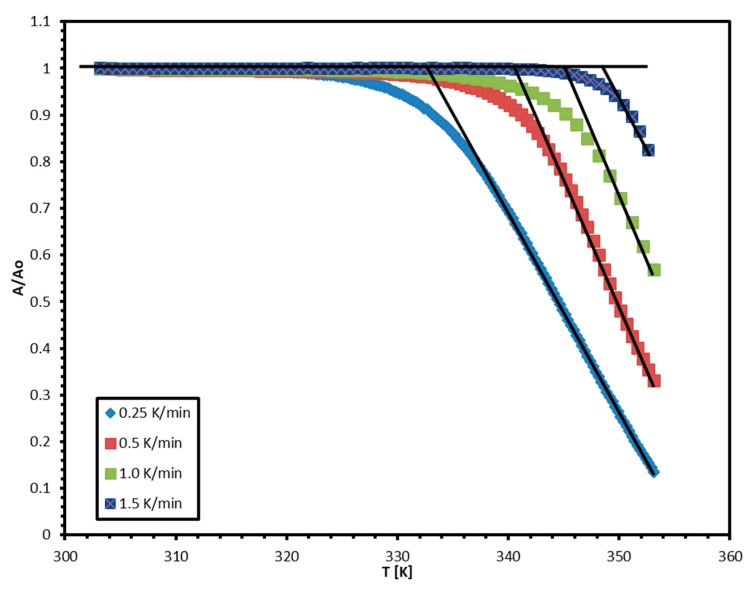
The effect of heating rate on the absorbance non-isotherms of TNT.

Figure 2 shows the normalized non-isothermal absorbance change in TNT monitored at the 264 nm peak upon heating at four different heating rates from 0.25 K/min to 1.5 K/min. The heating effect on the non-isotherms of TNT is clearly observed and consistent. The sublimation process is a single step process in the defined temperatures ranges (30–80 °C). For a specific weight loss, the sublimation temperature rises by increasing the heating rate. The non-isotherms show that the change in absorbance is insignificant at the beginning of the heating process until the temperature reaches a specific temperature known as the onset temperature (*T_o_*). The onset temperature (*T_o_*) can be determined as the temperature at which the line of best fit for the linear part of each isotherm intersects with the zero mass loss line (zero absorbance change line in Figure 2). Each isotherm shows a different onset temperature (*T_o_*) that increases with increasing the heating rate.

**Figure 3:**
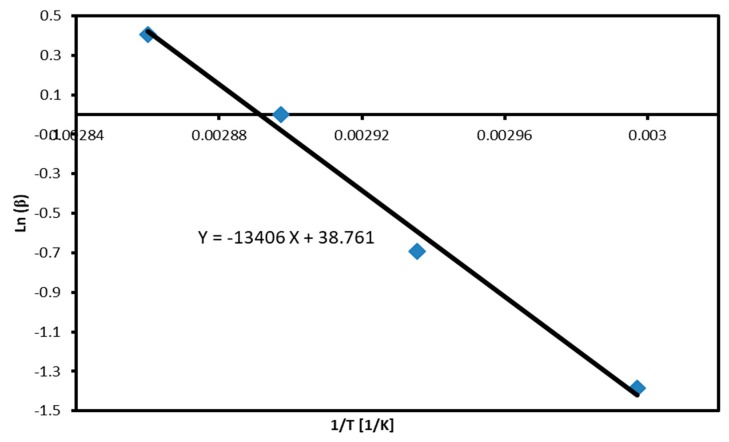
Plot of *ln(β)* versus the inverse of the absolute onset temperatures according to the Ozawa method.

Figure 3 shows a plot of *ln(β)* versus the inverse of the absolute onset temperatures according to the Ozawa method. The plot is linear and the activation energy is calculated to be 105.9 ± 1.4 KJ/mol. 

**Figure 4:**
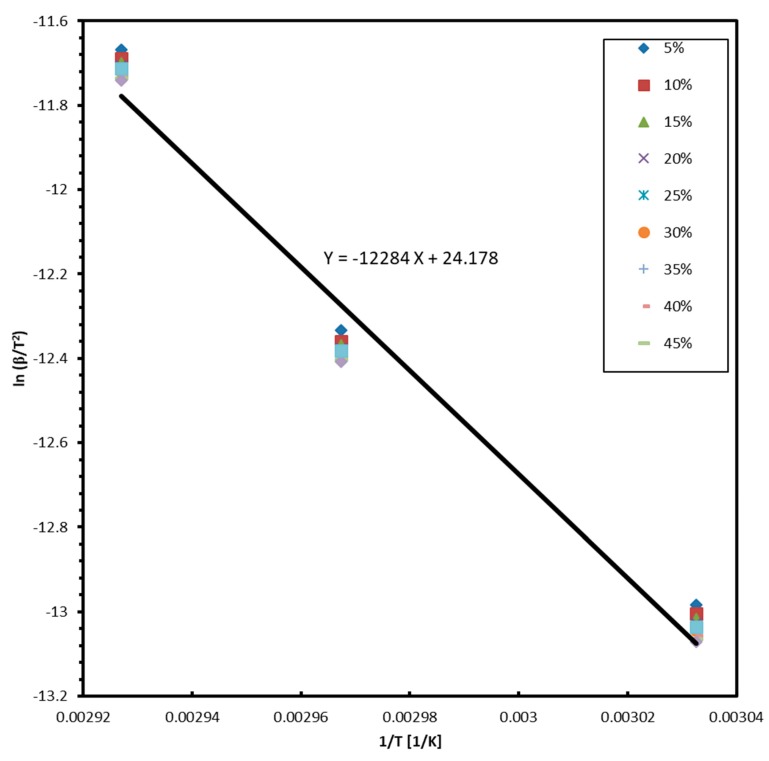
Plot of $ln\left(\beta /T^{2}\right)$ versus the inverse of the absolute temperature with weight loss from 0.05 to 0.5 in steps of 0.05 according to Flynn–Wall method (β = 0.25, 0.5, 1.0 K/min).

Figure 4 shows a plot of *ln(β/T^2^)* versus the inverse of the absolute temperatures corresponding to specific weight losses ranging from 5 to 50% for three heating rates, according to the Flynn–Wall method. The plots are linear and result in very close activation energies of sublimation values. However, for clarity, only the linear fit (black line) for the average values is shown. The activation energy was calculated to be 102.1 ± 2.7 KJ/mol.

**Figure 5:**
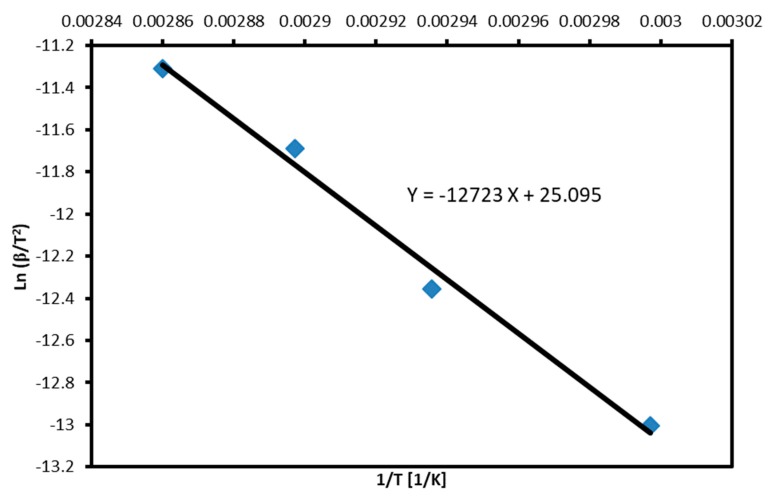
Plot of $ln\beta /T_{o}^{2}$ versus the inverse of the absolute temperature according to Kissinger method.

Figure 5 represents a plot of *ln(β/T^2^)* versus the inverse of the absolute onset temperatures according to the Kissinger method. The plot is linear and the activation energy was calculated to be 105.8 ± 1.6 KJ/mol. 

The results indicate good agreement between the values calculated using the Ozawa method, Flynn-wall method, and Kissinger method.

There is a large discrepancy (90–141 kJ/mol) in the activation energies described for TNT sublimation in literature [4,5,21,22,23,24,25,26,27]. The values measured here are in very good agreement with the values determined by the most sensitive techniques used in measuring sublimation kinetics of TNT: QCM and Knudsen effusion method 97 ± 7 and 103 kJ/mole, respectively [4,23]. In addition, the value reported here is in good agreement with the value determined isothermally using UV-Vis spectroscopy, 99.6 ± 5 kJ/mole [8]. The results illustrates that the UV-Vis absorbance spectroscopy can be used in the same manner as TGA and DSC to non-isothermally measure the activation energy of sublimation for low volatile materials, aided by the most common analytical techniques.

## 5. Conclusions

TNT nanofilms’ sublimation kinetics was investigated using UV-Vis absorbance spectroscopy in the UV range. Non-isothermal heating of the nanofilms at different heating rates was used in this study to determine the activation energy for TNT sublimation. Increasing the heating rate increases the onset temperature of sublimation observed by the technique as well as the temperature corresponding to a specific mass loss. The common data collection procedure followed in common thermal analysis techniques was used in this work with absorbance spectroscopy. Three different non-isothermal analysis models were used and the activation energy for TNT sublimation was calculated. The values are in excellent agreement, and agree well with the values reported using QCM and isothermal UV-Vis absorbance spectroscopy. This method, with the validated new data collection procedure, is expected to have a high impact on the popularity of the new technique and on the field of volatile hazardous materials’ detection.
